# Esophageal adenosquamous carcinoma with mucinous carcinoma-predominant lymph node metastases: a case report

**DOI:** 10.1186/s44215-026-00269-y

**Published:** 2026-08-25

**Authors:** Natsumi Maeda, Miho Yamamoto, Rie Nakashima, Kohei Tajima, Takatoshi Seki, Mika Ogimi, Kohei Kanamori, Yamato Ninomiya, Akihito Kazuno, Yoshiaki Shoji, Hiroshi Kajiwara, Masaki Mori, Kazuo Koyanagi

**Affiliations:** 1 Department of Gastroenterological Surgery, Tokai University School of Medicine, Isehara, Japan; 2 Department of Pathology, Tokai University School of Medicine, Isehara, Japan

**Keywords:** Adenosquamous carcinoma, Esophageal cancer, Mucinous carcinoma

## Abstract

**Background:**

Esophageal adenosquamous carcinoma is a rare histological subtype of esophageal cancer characterized by the coexistence of squamous cell carcinoma and adenocarcinoma components. Preoperative diagnosis is challenging because biopsy specimens often contain only the squamous component.

**Case presentation:**

A 75-year-old man presented with dysphagia. Esophagogastroduodenoscopy revealed a type 3 tumor in the middle thoracic esophagus. Histopathological examination of the biopsy specimens demonstrated predominantly squamous cell carcinoma with a minor mucin-producing adenocarcinoma component, raising suspicion of adenosquamous carcinoma. Given the patient’s history of myocardial infarction and poor cardiac function, neoadjuvant chemotherapy was considered unsuitable. The patient underwent thoracoscopic esophagectomy with lymph node dissection. Pathological examination confirmed adenosquamous carcinoma, with the superficial portion composed predominantly of moderately differentiated squamous cell carcinoma, whereas the deeper layers contained mucinous carcinoma and signet-ring cells. Extensive lymph node metastases were identified in the cervical, thoracic, and abdominal regions, the majority of which predominantly comprised mucinous carcinoma.

At seven months postoperatively, mediastinal lymph node recurrence was detected. Due to the patient’s deteriorating general condition, systemic chemotherapy was contraindicated, and the patient died 11 months postoperatively.

**Conclusions:**

We report a rare case of esophageal adenosquamous carcinoma with mucinous carcinoma-predominant lymph node metastases. The predominance of mucinous carcinoma in metastatic lesions may reflect the aggressive biological behavior of the adenocarcinoma component and could be associated with poor clinical outcomes. Although esophageal adenosquamous carcinoma is generally managed using surgery-based multidisciplinary strategies based on the treatment principles for esophageal squamous cell carcinoma, no standard chemotherapy regimen has been established for esophageal adenosquamous carcinoma. Further accumulation of cases is needed to determine the optimal treatment strategies for this rare disease.

## Background

Esophageal adenosquamous carcinoma (ASC) is a rare histological subtype of esophageal cancer characterized by the coexistence of squamous cell carcinoma and adenocarcinoma components. ASC accounts for approximately 0.2–1.0% of all esophageal malignancies [1, 2]. The squamous component typically comprises the tumor's superficial layer, whereas the deeper adenocarcinoma component is frequently missed in biopsy specimens, thereby complicating preoperative diagnosis [2]. The optimal treatment strategy for esophageal ASC remains unclear.

Given its rarity, the biological behavior and clinical characteristics of this malignancy remain poorly understood. The adenocarcinoma component may drive tumor progression and metastasis [3]. However, cases in which the mucin-producing adenocarcinoma component predominates in metastatic deposits are extremely rare.

Herein, we report a case of esophageal ASC with mucinous carcinoma-predominant lymph node metastases.

## Case presentation

**Fig. 1:**
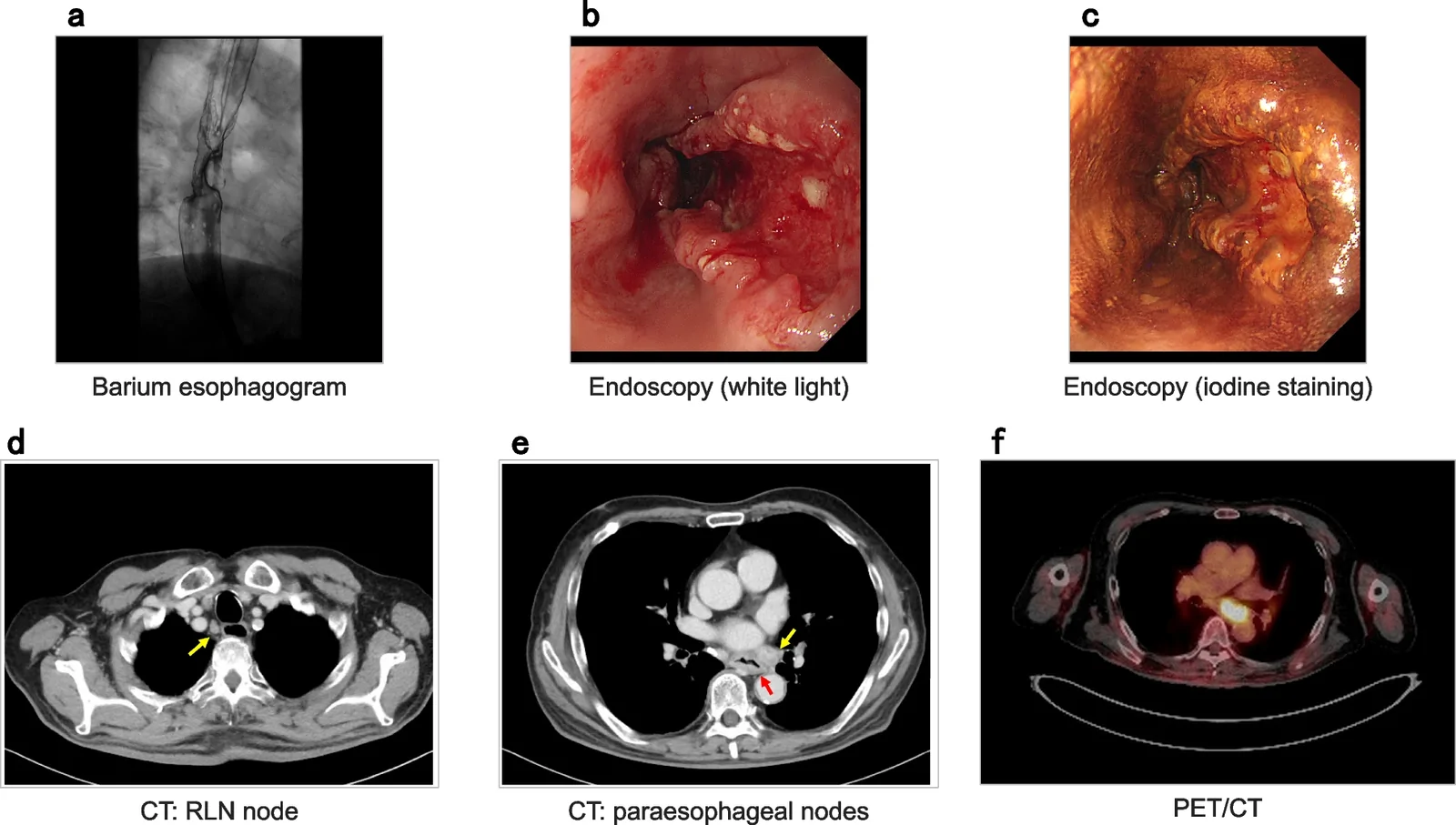
Preoperative imaging. **a** Barium esophagography showing an ulcerative lesion extending from the upper to the middle thoracic esophagus. **b**, **c** Esophagogastroduodenoscopy showing a type 3 advanced tumor located 25–30 cm from the incisors: (**b**) white-light imaging; (**c**) after iodine staining. **d**, **e** Contrast-enhanced computed tomography: (**d**) an enlarged lymph node along the right recurrent laryngeal nerve (RLN) (yellow arrow); (**e**) esophageal wall thickening in the middle thoracic esophagus (red arrow) and enlarged middle thoracic paraesophageal lymph nodes (yellow arrow). **f** Positron emission tomography (PET)-computed tomography showing fluorodeoxyglucose uptake extending from the upper to the middle thoracic esophagus (maximum standardized uptake value, 11.6)

**Fig. 2:**
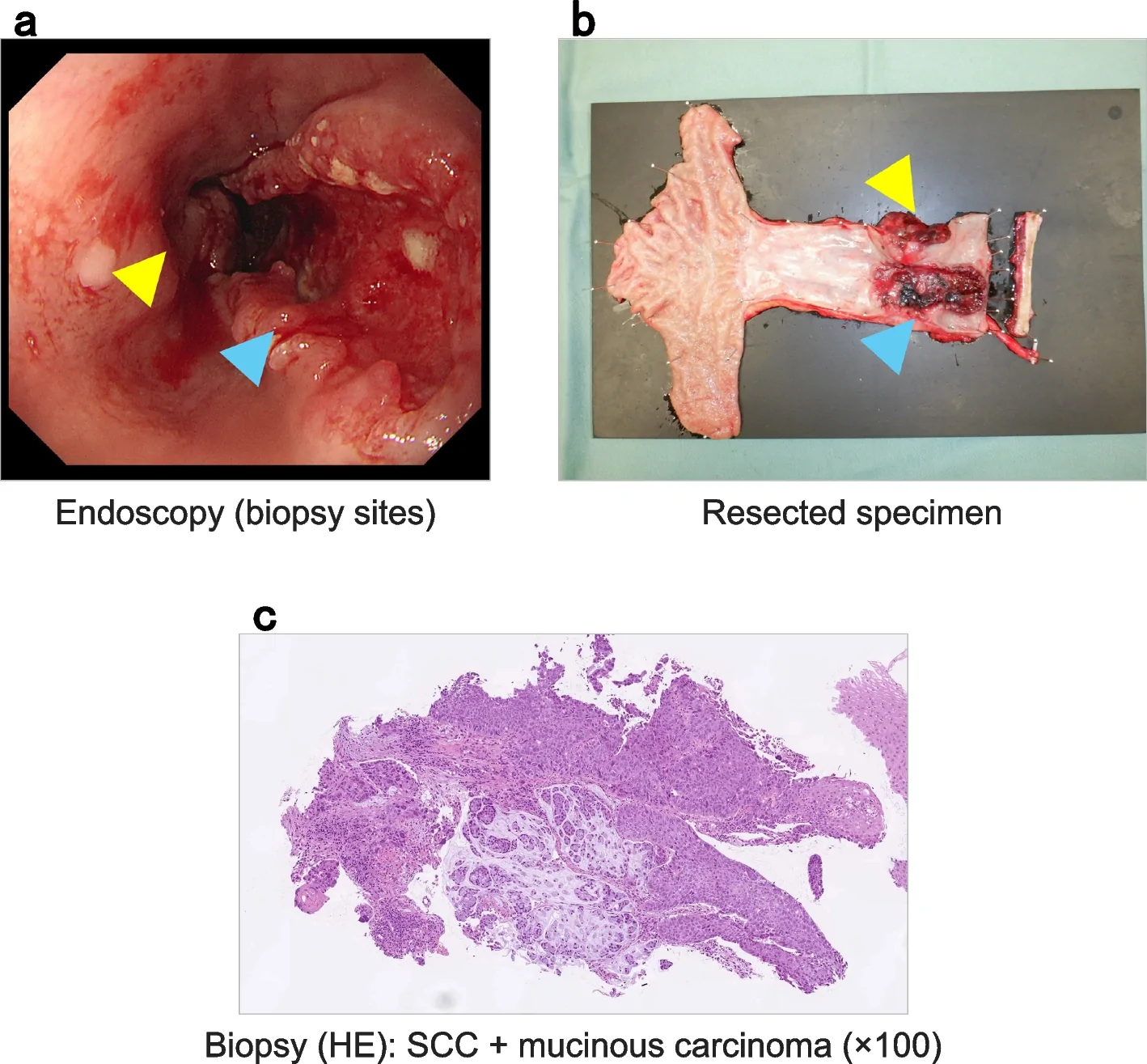
Correspondence between the preoperative biopsy sites and the resected specimen. **a** Endoscopy showing the two biopsy sites: the oral Type 2 lesion (blue arrow), which yielded squamous cell carcinoma (SCC), and the anal elevated lesion (yellow arrow), which yielded squamous cell carcinoma and mucinous carcinoma. **b** Resected specimen; arrows of the same color indicate the corresponding sites. The elevated lesion (yellow arrow) corresponded to a metastatic lymph node directly invading the esophageal wall. **c** Histopathology of the biopsy specimen from the elevated lesion, showing squamous cell carcinoma with a mucinous carcinoma component [hematoxylin and eosin (HE) staining, × 100]

A 75-year-old man was referred to our hospital with dysphagia. He had a significant smoking history and a medical history of diabetes mellitus, myocardial infarction, prostate cancer, and bladder cancer. Physical examination yielded unremarkable findings. The carcinoembryonic antigen (CEA) level was elevated at 13.6 ng/mL, while other laboratory parameters were within normal limits. Barium esophagography identified an ulcerative lesion extending from the upper to the middle thoracic esophagus (Fig. 1a). Esophagogastroduodenoscopy revealed a type 3 advanced tumor located 25–30 cm from the incisors (Fig. 1b, c). Endoscopic biopsies were obtained from two sites (Fig. 2a). The oral type 2 portion of the tumor (blue arrow) demonstrated squamous cell carcinoma, whereas the anal lesion, which appeared elevated (yellow arrow), demonstrated both squamous cell carcinoma and mucinous carcinoma components (Fig. 2c), raising the suspicion of ASC. Contrast-enhanced computed tomography revealed thickening of the esophageal wall, along with enlargement of the right recurrent laryngeal nerve and middle thoracic paraesophageal lymph nodes (Fig. 1d, e). No distant metastases were identified. Positron emission tomography-computed tomography (PET-CT) revealed fluorodeoxyglucose uptake in the upper-to-middle thoracic esophagus, with a maximum standardized uptake value of 11.6; however, no abnormal fluorodeoxyglucose uptake was identified in the metastatic regional lymph nodes (Fig. 1f). The patient was diagnosed with clinical stage III (cT3N2M0) esophageal cancer with suspected ASC. Due to the patient’s history of myocardial infarction and poor cardiac function, neoadjuvant chemotherapy was deemed unsuitable. He underwent thoracoscopic esophagectomy with laparoscopic gastric tube reconstruction, three-field lymph node dissection, and jejunostomy.

**Fig. 3:**
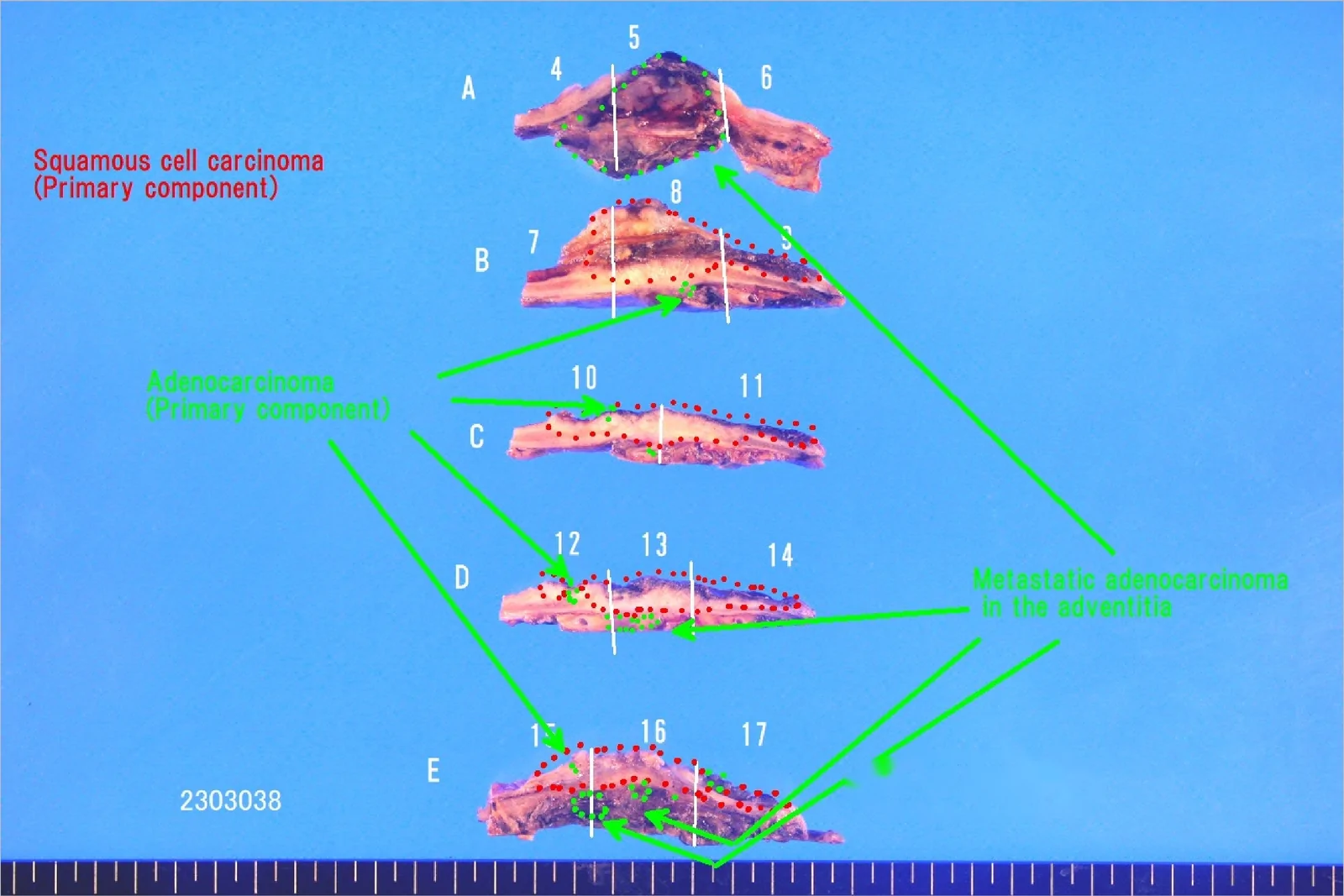
Mapping of histopathological findings on the cut surface of the resected specimen. The superficial layer consisted of squamous cell carcinoma, whereas adenocarcinoma components were identified on the adventitial side, corresponding to tumor invasion from metastatic lymph nodes

**Fig. 4:**
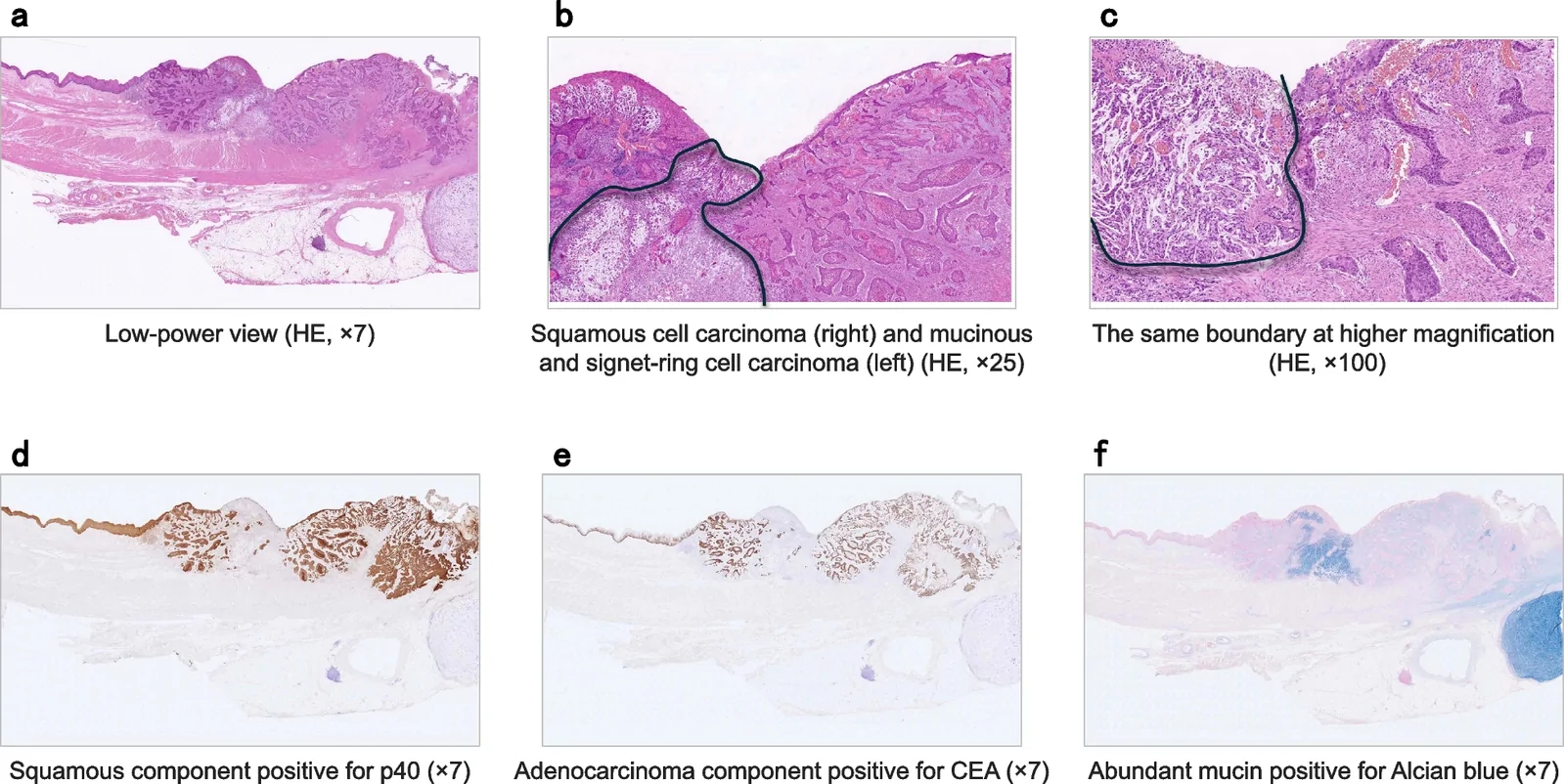
Histopathology and immunohistochemistry of the resected tumor. **a** Low-power view showing squamous, glandular, and mucinous components (hematoxylin and eosin staining, × 7). **b**, **c** Higher-power views of the boundary between squamous cell carcinoma and mucinous carcinoma containing signet-ring cells (hematoxylin and eosin staining; b, × 25; c, × 100). **d** Squamous cell carcinoma component positive for p40 (× 7). **e** Adenocarcinoma component positive for carcinoembryonic antigen (× 7). **f** Abundant mucin positive for Alcian blue (× 7)

**Fig. 5:**
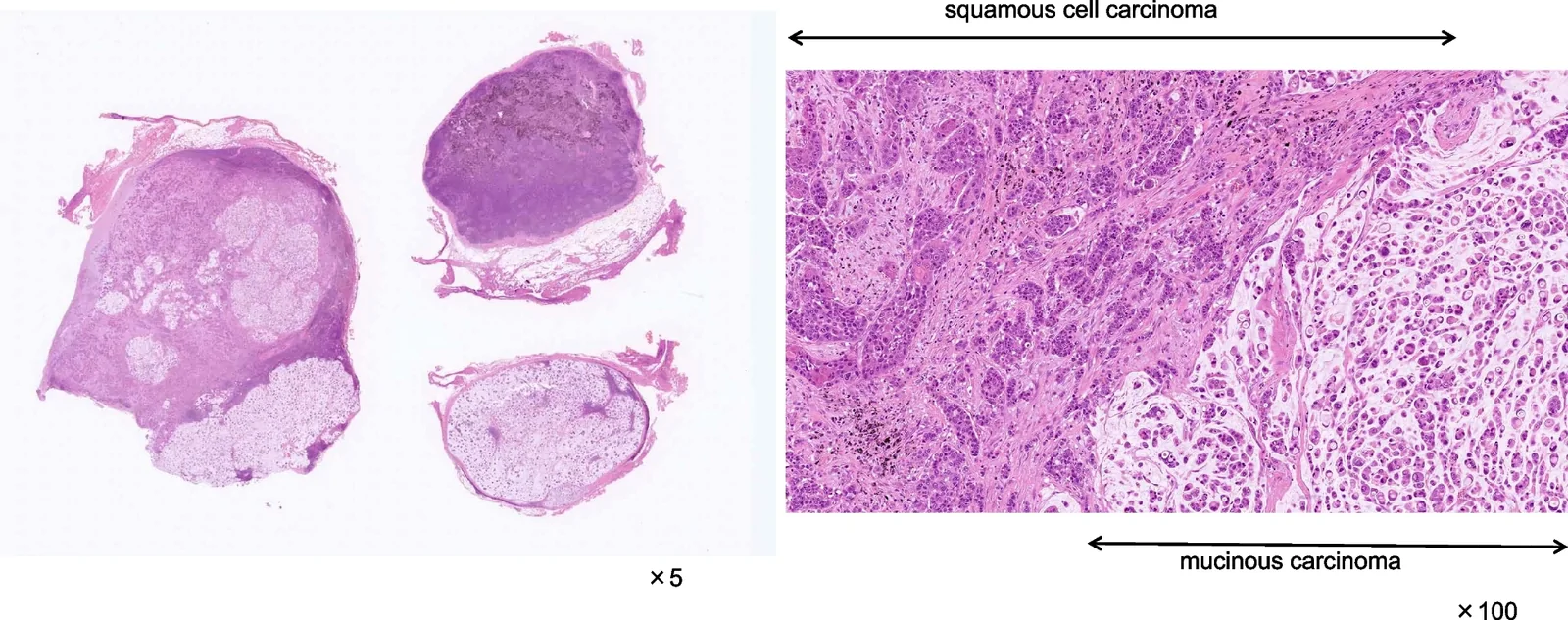
Histopathology of the metastatic lymph nodes (hematoxylin and eosin staining). Low- (× 5) and high-power (× 100) views showing that the majority of the metastatic lesion consists of mucinous carcinoma with a minor squamous cell carcinoma component

Histopathological examination of the resected specimen revealed that the majority of the tumor consisted of squamous cell carcinoma, with a minor mucin-producing adenocarcinoma component in the deeper layers (Fig. 3). The superficial portion comprised moderately differentiated squamous cell carcinoma, whereas the deeper layers contained mucinous carcinoma with abundant mucin and signet-ring cells, with transitional features between the two (Fig. 4a–c). Immunohistochemical staining confirmed squamous, glandular, and mucinous components, establishing the diagnosis of ASC (Fig. 4d–f). Overall, 18 of 46 dissected lymph nodes were metastatic (pN3), and involved the cervical, thoracic, and abdominal fields (Table 1). The nodal metastases were composed predominantly of mucinous carcinoma; in most involved stations, the metastatic tissue was purely or predominantly mucinous (approximately 60–100% of the deposit). A squamous cell carcinoma component was identified in only one station (the main bronchus node, comprising about 25% of that deposit), and one small abdominal node (lesser curvature) contained pure tubular adenocarcinoma (Fig. 5). Lymphatic and vascular invasion was almost entirely composed of the mucinous carcinoma component; although the squamous cell carcinoma component also produced nodal metastases, no definite vascular invasion by the squamous component was identified. In the resected specimen, the elevated lesion that had yielded the mucinous component on biopsy corresponded to a metastatic lymph node directly invading the esophageal wall, where the adenocarcinoma component was exposed to the luminal surface (Fig. 2b). The final diagnosis was stage IVA (pT3N3M0) ASC.

**Table 1:** Histological composition of lymph node metastases by station

Region	Station	Metastatic/ dissected nodes	Predominant component	Histological composition (%)
Cervical	101R/L	3/4	Mucinous	Mucinous 60, tubular 40
	104R/L	0/16	—	—
Thoracic	105	4/4	Mucinous	Mucinous 70, tubular 30
	106recR	3/4	Mucinous	Mucinous 95, tubular 5
	106recL	2/2	Mucinous	Mucinous 100
	107	1/1	Mucinous	Mucinous 100
	108ᵃ	2/3	Mucinous	Mucinous 80, tubular 20
	109L	2/3	Mucinous	Mucinous 70, SCC 25, tubular 5
Abdominal	1・2	0/3	—	—
	3	1/1	Adenocarcinoma (tubular)	Tubular 100
	7	0/5	—	—
Total		18/46	Mucinous-predominant	pN3

*Abbreviations*: *LN* lymph node, *SCC* squamous cell carcinoma. Station numbers follow the Japanese Classification of Esophageal Cancer. Percentages indicate the approximate semi-quantitative proportion of each component within the metastatic deposit. Lymphatic and venous invasion was almost exclusively composed of the mucinous carcinoma component; the squamous cell carcinoma component produced nodal metastases but showed no definite vascular invasion. ᵃStation 108 corresponded to the metastatic node that directly invaded the esophageal wall (see text and Fig. 2)

Postoperatively, the patient’s performance status deteriorated; therefore, adjuvant chemotherapy was withheld. At seven months postoperatively, CEA levels increased, and mediastinal lymph node recurrence was detected. Given the worsening general condition, systemic chemotherapy remained contraindicated, eventually resulting in his death 11 months postoperatively.

## Discussion

The present case report on esophageal ASC was distinctive in two respects: the adenocarcinoma component was captured on preoperative biopsy, and the lymph node metastases were predominantly composed of mucinous rather than squamous carcinoma. Considered alongside the differential diagnosis and published literature, these findings indicate that the adenocarcinoma component can dominate the metastatic behavior of this rare tumor.

Mucoepidermoid carcinoma (MEC), a distinct epithelial tumor composed of squamous, mucus-secreting, and intermediate cells, is a distinct entity from ASC in the World Health Organization classification and should be considered in the differential diagnosis [3, 4]. In the present case, both squamous cell carcinoma and gland-forming adenocarcinoma components were identified, supporting ASC rather than MEC.

To ascertain the clinicopathological characteristics of esophageal ASC, we reviewed individual case reports published in PubMed over the past two decades, excluding case series, database studies, and review articles. Four cases, including the present case, were identified, with variable tumor locations (upper or lower thoracic esophagus and the esophagogastric junction) and macroscopic appearance (type 2, type 3, or submucosal tumor-like) (Table 2). In all cases except the present one, the preoperative diagnosis was squamous cell carcinoma, highlighting the difficulty in identifying adenocarcinoma components in biopsy specimens [5–7]. Surgery was performed in most cases, with adjuvant chemoradiotherapy or oral S-1 in two. Three single-institution retrospective studies have also been reported [2, 8, 9] (Table 3). The preoperative diagnostic rate was low; in the two series that reported it, adenosquamous carcinoma was correctly identified before surgery in only three of 38 [8] and one of 43 [9] patients, respectively. Survival outcomes varied substantially among these series, with reported 5-year overall survival rates ranging from approximately 30% to 64%. Although esophageal ASC has historically been regarded as aggressive, the largest surgical series with propensity-score matching found that overall survival after esophagectomy was comparable to that of esophageal squamous cell carcinoma [9], whereas other reports described less favorable outcomes [8]. Collectively, these data suggest that the prognosis of esophageal ASC is at least as unfavorable as that of squamous cell carcinoma and is strongly influenced by nodal status and the completeness of resection, rather than being uniformly poor.

**Table 2:** Summary of case reports of esophageal adenosquamous carcinoma

Author	Year	Country	Age/sex	Tumor location	Macroscopic type	LN metastasis (N)	Histology of LN metastases	Preoperative diagnosis	Treatment	Chemotherapy/adjuvant therapy	Outcome	Ref
Francioni et al	2012	Italy	75/M	Lower thoracic/EGJ	Ulcerating tumor	Yes; pN1 (pT3N1M0)	NR	SCC	Surgery	Yes; adjuvant chemoradiotherapy	Died 24 months after surgery; mediastinal recurrence and distant metastases	5
Matsukuma et al	2017	Japan	53/M	Upper thoracic	Ulcerating tumor	Yes; autopsy, widespread (incl. LN)	Adenocarcinoma (discohesive) component	SCC	No surgery	No cancer-directed chemotherapy before death	Died 1 month after first hospitalization; autopsy case	6
Fukai et al	2022	Japan	81/M	EGJ	SMT-like elevated tumor	Yes; pN3 (8/32 nodes)	SCC + adenocarcinoma (7/8 nodes)	SCC	Surgery	Yes; adjuvant oral S-1; no neoadjuvant chemotherapy	Alive without recurrence 6 months after surgery	7
Present case	2024	Japan	75/M	Middle thoracic	Type 3	Yes; pN3 (18/46 nodes)	Mucinous carcinoma-predominant	ASC suspected	Surgery	No	Died 11 months after surgery	—

*Abbreviations*: *AC* adenocarcinoma, *ASC* adenosquamous carcinoma, *CRT* chemoradiotherapy, *EGJ* esophagogastric junction, *LN* lymph node, *NAC* neoadjuvant chemotherapy, *NR* not reported, *SCC* squamous cell carcinoma, *SMT* submucosal tumor. N stage follows the AJCC/UICC TNM classification. Treatment was simplified as surgery or no surgery; details of operative procedures were intentionally omitted for table readability

**Table 3:** Summary of single-institution retrospective series of esophageal adenosquamous carcinoma

Author	Year	Cases	Treatment	Surgery	Chemotherapy/adjuvant therapy	Histology of LN metastases	Preoperative diagnosis	Outcome	Ref
Yachida et al	2004	18	Surgery-based multidisciplinary treatment	Esophagectomy with LND	Chemotherapy NR; preoperative RT excluded	NR	NR	5-yr OS 63.6%; 10-yr OS 47.7%	2
Ni et al	2016	39	Surgery-based multidisciplinary treatment	Esophagectomy with LND	NAC 1; adjuvant RT 3; CT 6; CRT 6	NR	3/38 (7.9%)	MST 44.4 mo; 5-yr OS 37.5%	8
Chen et al	2022	56	Surgery-based multidisciplinary treatment	Esophagectomy with LND	No NAC; adjuvant RT 11; CT 5; CRT 3	NR	1/43 (2.3%)	MST 32.0 mo; 5-yr OS 29.6%	9

*Abbreviations*: *CT* chemotherapy, *CRT* chemoradiotherapy, *LND* lymph node dissection, *MST* median survival time, *NAC* neoadjuvant chemotherapy, *NR* not reported, *OS* overall survival, *RT* radiotherapy. The preoperative diagnosis column indicates the number of patients correctly diagnosed as adenosquamous carcinoma on preoperative biopsy, with the remainder mostly misdiagnosed as squamous cell carcinoma. The histological composition of lymph node metastases was not characterized in these institutional series

In contrast to most reported cases, ASC was suspected preoperatively in this patient because of an unusual tumor distribution: a metastatic lymph node containing abundant mucinous carcinoma had directly invaded the esophageal wall and exposed the adenocarcinoma component to the lumen (Fig. 2). Because the deep adenocarcinoma component is usually inaccessible to forceps biopsy, this direct luminal exposure allowed both components to be captured preoperatively.

Detailed nodal mapping confirmed that the metastatic burden was driven by the adenocarcinoma (mucinous) component: mucinous carcinoma predominated in virtually all involved stations and accounted for almost all lymphatic and vascular invasion, whereas the squamous component, although capable of nodal metastasis, showed no definite vascular invasion. This dissociation between the squamous cell carcinoma-predominant primary lesion and the mucinous carcinoma-predominant metastases supports the view that the adenocarcinoma component governs the aggressive metastatic behavior of esophageal ASC. Notably, in previously reported esophageal ASC, nodal metastases predominantly comprised the squamous component (60.9–85.7%), whereas the adenocarcinoma component alone accounted for only 0–8.7% [7]; the mucinous carcinoma-predominant metastases in the present case are therefore exceptional.

This mucinous predominance also affects preoperative imaging. Although the primary tumor was intensely fluorodeoxyglucose-avid (maximum standardized uptake value, 11.6), the metastatic lymph nodes showed no abnormal uptake on PET-CT. Since mucinous carcinoma is frequently poorly fluorodeoxyglucose-avid, PET-CT may underestimate nodal metastases in mucinous carcinoma-predominant esophageal ASC, and this limitation should be considered during preoperative staging.

Mucinous adenocarcinoma is rare among esophageal cancers and is characterized by abundant extracellular mucin production [10]. Mucin production may promote tumor invasion and metastasis by reducing intercellular adhesion and increasing tumor cell mobility [11–13], and mucinous carcinomas of the colorectum and stomach are similarly associated with advanced disease and lymph node metastasis [14, 15]. These findings suggest that the mucin-producing component may have contributed to the aggressive metastatic behavior observed in the present case.

No standard systemic therapy has been established for esophageal ASC, and treatment generally follows the principles used for esophageal squamous cell carcinoma within a surgery-based multidisciplinary strategy, including immune checkpoint inhibitor-based regimens for advanced disease [16, 17]. Because our patient received no systemic therapy, conclusions regarding chemotherapy sensitivity cannot be drawn. Mucinous carcinomas in other organs are often less responsive to conventional chemotherapy [18], possibly because mucin impairs drug delivery [19]; biomarker evaluation and regimens used for esophagogastric adenocarcinoma [20] may therefore be considered in selected cases.

## Conclusions

We report a rare case of esophageal ASC with mucinous carcinoma-predominant lymph node metastases, in which the mucin-producing adenocarcinoma component appeared to drive the aggressive metastatic behavior. Treatment has not been established and is generally surgery-based and multidisciplinary, following the principles used for esophageal squamous cell carcinoma. Further accumulation of cases with detailed treatment and biomarker data is required to define optimal therapy.

## Data Availability

Data sharing is not applicable to this article because no datasets were generated or analyzed during the current study.

## Abbreviations

ASC: Adenosquamous carcinoma
CEA: Carcinoembryonic antigen
PET-CT: Positron emission tomography-computed tomography
MEC: Mucoepidermoid carcinoma
